# Frequency of Amblyopia in strabismus patients presenting to tertiary care hospital

**DOI:** 10.22336/rjo.2023.8

**Published:** 2023

**Authors:** Khalil Khan Zahir, Muhammad Israr, Muhammad Adnan Khan Khattak, Saman Mudassar, Samia Shaheen, Irfan Ullah

**Affiliations:** * Hayatabad Medical Complex (HMC) Peshawar, Pakistan; ** Health Department, Khyber Pakhtunkhwa, Pakistan; *** Combined Military Hospital (CMH) Peshawar, Pakistan

**Keywords:** amblyopia, squint, strabismus

## Abstract

**Objective:** The rationale of study was to find the magnitude of amblyopia with reference to type of squint among the strabismus patients visiting Hayatabad Medical Complex Peshawar, Pakistan.

**Materials and Methods:** After ethical approval, a cross sectional study was carried out in the Department of Ophthalmology, Hayatabad Medical Complex, Peshawar, Pakistan, from April 2022 to October 2022, the total number of patients included being 237.

**Results:** Amblyopia was observed in 113 out of 160 (70.6%) cases of uniocular squint, while in alternating squint it was found to be 11 out of 77 (14.2%). Amblyopia in patients with esotropia was seen in 73.2% (107 out of 146), while 59.3% (54 out of 91) exotropia had associated amblyopia.

**Conclusion:** Strabismus amblyopia leads to developmental arrest of vision in early critical years of life. Permanent visual loss can be avoided with comprehensive screening and detailed examination of strabismic patient.

## Introduction

Reduced vision in one eye (amblyopia) is caused by faulty visual development early in infancy. The weaker (or lazy) eye is prone to wandering inward or outward. Amblyopia is a condition that affects people from infancy to the age of seven. It is the most common cause of eyesight loss in children. Lazy eye rarely affects both eyes [**1**].

A squint, also known as strabismus, is a condition of misalignment of the eyes, and if it is not properly treated, it effects binocularity and depth perception. It is more prevalent in young children, although it can happen to anyone at any age. One eye may turn in, out, up, or down, while the other gazes forward [**2**].

Heredity, eye muscle weakness or an issue with the nerves in the eye muscles, cataracts, glaucoma, corneal scars, optic nerve illness, refractive errors, eye tumors, injuries and retinal disease, among other things, can substantially impair one’s eyesight causing squint [**3**].

Amblyopia develops when there is a significant disparity in the capacity to focus between the two eyes. Other vision issues are the most common cause of amblyopia. It is critical to address these other issues, or the brain will begin to rely on the eye with a better vision, resulting in amblyopia [**4**].

Commonly, amblyopia is of either refractory, strabismic, sensory deprivation, or of meridional type [**5**,**6**].

Early aberrant visual experience in children can disrupt interocular alignment, causing strabismus, interfere with sensory development, causing amblyopia, and change the path of emmetropization, causing ametropias in one or both eyes. Given that each of these disorders has the ability to modify visual perception, the existence of any one of these conditions in early life could cause one or both of the other two. In this regard, amblyopia is significantly linked to the occurrence of anisometropia and/ or strabismus during early childhood [**7**]. The rationale of the study is to find the magnitude of amblyopia with reference to type of squint among the strabismus patients visiting the Ophthalmology Department of Hayatabad Medical Complex, Peshawar, Pakistan, so that the strategies could be made for early diagnosis and prompt treatment. As no reliable data is available regarding the frequency of amblyopia in the strabismus patients in Khyber Pakhtunkhwa, this study will contribute to the future planning regarding strabismus Amblyopia management.

## Material and methods

A cross sectional study was carried out in the Department of Ophthalmology, Hayatabad Medical Complex, Peshawar, Pakistan, from April 2022 to October 2022, after the approval of the Ethical Committee. Patients who presented to the Outpatient Department with squint were screened for amblyopia. The sample size was calculated using WHO sample size formula with 95% confidence interval, 3% margin of error and expected frequency of amblyopia by 19% in patients with strabismus (squint) and was 237 [**8**]. Non-probability consecutive sampling was used to enroll the patients. Patients of all ages, gender and types of squints were included, while patients with previous history of ocular trauma, surgery or any neurological disease were excluded. Base line demographic information of patients (age, gender, duration of complain) were considered. Informed consent was obtained from parents/ care givers, patients themselves, ensuring confidentiality, and explaining the risk and benefit involved to the patients who took part in this study. All the patients who met the inclusion criteria, with squint, were assessed using a standard approach that included a medical history, visual acuity (Snellen chart), orthoptic assessment, silt lamp biomicroscopy and indirect fundoscopy. Frequency of amblyopia was noted on an especially designed proforma. Amblyopia was further graded into mild and moderate and dense categories. All the recorded data was analyzed using IBM-SPSS version 23. Quantitative variables like age and duration of complain were measured as mean and standard deviation. Qualitative variables like gender, side of eye, single or bilateral eye involvement were measured in terms of frequency percentages. Factors were stratified by age, gender, side of eye and type of squint and grade of amblyopia. Post stratification chi square test was applied, p ≤ 0.05 being considered statistically significant. 

## Results

**Fig. 1:**
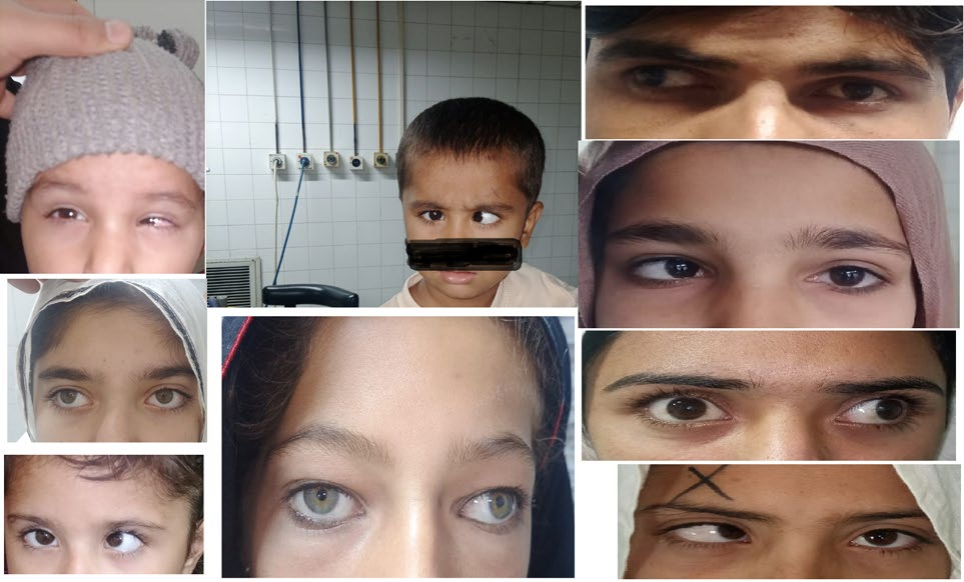
Different types of squint with associated amblyopia

Two hundred and thirty-seven (237) patients in total participated in the study. The patients’ ages ranged from 1 year to 63 years. Males were found to be less prevalent than females (46% vs. 54%). 160 (67.5%) cases were registered with uniocular squint (right eye affected in 93 and left eye in 67 cases). 77 (44%) cases were registered with alternating squint (**Fig. 1**).

In 76 cases, the best corrected visual acuity was 6/ 6 in both eyes. Exotropia was seen in 91 (38.3%), while esotropia was observed in 146 (61.6%) cases. 

**Fig. 2:**
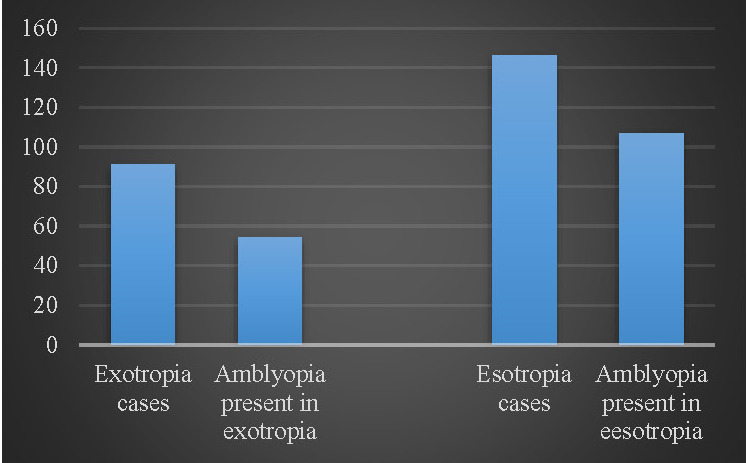
Amblyopia frequency in esotropia and exotropia

Amblyopia was observed in 113 out of 160 (70.6%) cases of uniocular squint, while in alternating squint, it was 11 out of 77 (14.2%). A total of 161 patients got any grade of amblyopia with strabismus (67%). In esotropia, amblyopia was observed in 73.2% (107 out of 146), while exotropia was observed in 59.3% (54 out of 91) and had associated amblyopia as shown in **Fig. 2**.

Amblyopia was further graded as mild, moderate and dense type. Moderate amblyopia was found in 78 (48.4%) cases, while mild in 54 (33.5%) and dense in 29 (18%) were less common. The overall prevalence was found to be 84.6% in both forms of squint (**Table 1**).

**Table 1:** Characteristics of amblyopia

CHARACTERISTICS OF AMBLYOPIA			
GENDER			
Male		46%	
Female		54%	
SQUINT			*Amblyopia Observed*
*Uniocular*	160	67.50%	113 (70.6%)
Right eye involvement	93		
Left eye involvement	67		
*Alternating*	77	44%	11 (14.2%)
Grade of amblyopia			
Mild	54 (33.5%)		
Moderate	78 (48.4%)		
Dense	29 (18%)		

## Discussion

Hashemi et al. [**9**] assessed strabismus, exotropia and esotropia to have a pooled prevalence of 1.93%, 1.23%, and 0.77%, respectively, and our study differed in the said prevalence due to our preset inclusion criteria for our study duly approved.

Another meta-analysis performed by Budan Hu et al. analyzed a total of 97 trials, involving 4,645,274 kids and 7,706 amblyopic patients. Amblyopia was present in 1.36% of people worldwide being generally of 95% CI: 1.27-1.46%. Males were more likely to experience amblyopia (OR = 0.885, 95% CI: 0.795-0.985, P = 0.025) than females (1.24%, 95% CI: 0.94-1.54%), our results slightly differing in gender distribution [**10**].

According to Dikova SP and col., 42 (2.5%) of the 1,675 children had amblyopia, of whom 3% had deprivation amblyopia, 59% had anisometropic amblyopia (25), 31% had isoametropic amblyopia (25), and 7% had strabismic amblyopia. 73% of the cases (27) had unilateral amblyopia, and 27% had bilateral amblyopia (15) [**11**]. The findings of our study corresponded closely to their observations.

Our results were also consistent with the description of childhood amblyopia provided by the Pediatric Eye Disease Investigator Group (PEDIG), which stated that the most common causes of amblyopia were strabismus and anisometropia. A quarter of the youngsters were also discovered to have components of both, such as strabismus and untreated refractive defects [**12**].

Our study results were parallel to what was found by Flom and Neumaier’s study of amblyopic kids in kindergarten through sixth grade, 38% of them having strabismus, 34% having anisometropia of 1 diopter or higher, and 28% having both strabismus and anisometropia. According to the results of the current investigation, anisometropia and strabismus are the main causes of unilateral amblyopia [**13**]. 

A dramatic decline in the prevalence of unilateral amblyopia among young adults, as well as the prevalence of current strabismus were found over the course of generation. The prevalence of severe (both unilaterally and bilaterally) strabismus remained steady. These changes coincided with the implementation of the national screening programme for children and the increased usefulness of amblyopia and strabismus treatments. The “natural history” of these diseases and their prevalence in adolescence may have been altered as a result of these early therapies [**14**]. 

## Conclusion

Strabismic amblyopia is a preventable condition if early attention and diagnosis is made.We need to implement screening programmes at school and Madrasa level to prevent lifelong visual impairment.

**Conflict of interest**

The authors state no conflict of interest.

**Informed Consent and Human and Animal Rights statement**

Informed consent has been obtained from all individuals included in this study.

**Authorization for the use of human subjects**

Ethical approval: The research related to human use complies with all the relevant national regulations, institutional policies, is in accordance with the tenets of the Helsinki Declaration, and has been approved by the review board of Hayatabad Medical Complex Peshawar, Pakistan.

**Acknowledgements**

None.

**Sources of Funding**

This study did not receive any financial grant from funding agencies in the public, commercial, or non-profit sectors.

**Disclosures**

None.
